# Remission Effects of Dietary Soybean Isoflavones on DSS-Induced Murine Colitis and an LPS-Activated Macrophage Cell Line

**DOI:** 10.3390/nu11081746

**Published:** 2019-07-29

**Authors:** Sang-Eun Kim, Koichiro Kawaguchi, Hiroko Hayashi, Katsuhiro Furusho, Mitsuo Maruyama

**Affiliations:** 1 Department of the Mechanism of Aging, National Center for Geriatrics and Gerontology 7-430 Morioka-Cho, Obu, Aichi 474-8511, Japan; 2 Department of Pathology, Nagasaki University School of Medicine, Graduate School of Biomedical Sciences, 1-12-4 Sakamoto, Nagasaki 852-8523, Japan; 3 Department of Aging Research, Nagoya University Graduate School of Medicine, 65 Tsurumai-cho, Showa-ku, Nagoya 466-8550, Japan

**Keywords:** Inflammatory bowel disease, dextran sodium sulfate, RAW264.7 macrophages, lipopolysaccharide, nuclear factor-kB, inducible nitric oxide, nitric oxide

## Abstract

Inflammatory bowel diseases (IBDs), including ulcerative colitis and Crohn’s disease, are chronic disorders of the gastrointestinal tract, although the exact causes of IBD remain unknown. Present treatments for IBDs have poor tolerability and insufficient therapeutic efficacy, thus, alternative therapeutic approaches are required. Soybean-derived isoflavones have multiple bioactivities such as anti-inflammation. However, the low water solubility of soybean isoflavones limits their bioavailability and practical use. Therefore, in order to study the preventive effects of water-soluble soybean isoflavones on colonic inflammatory status, we examined soybean-derived isoflavone glycosides (SIFs) in a dextran sodium sulfate (DSS)-induced murine colitis model and in lipopolysaccharide (LPS)-activated RAW264.7 macrophages. Oral administration of SIF (0.5 w/v%) attenuated DSS-induced colitis in terms of body weight decrease, colon shortening, epithelial apoptosis, histological score, mRNA levels of inflammatory cytokines, and immune cell infiltration in colon tissues. In the in vitro assessment, we observed the inhibitory effects of SIF on the production of nitric oxide and prostaglandin E2, via suppression of inducible nitric oxide synthase and cyclooxygenase-2 expression in RAW264.7 macrophages in response to LPS. Furthermore, we confirmed that the expression of inflammatory cytokines and chemokines were decreased by pre-treatment with SIF in LPS-activated RAW264.7 macrophages. Moreover, we demonstrated that SIF suppressed inflammatory mediators involved in nuclear factor-κB signaling pathway via inhibitory κB kinase phosphorylation and degradation of inhibitory κB. Our results suggested that SIF may be beneficial for the remission of colonic inflammatory status including IBDs.

## 1. Introduction

Inflammatory bowel diseases (IBDs), mainly Crohn’s disease and ulcerative colitis, are chronic relapsing inflammatory conditions characterized by an abnormal immune response to intestinal microflora, impaired epithelial barrier function, tissue damage, diarrhea, rectal bleeding, and abdominal pain [1,2,3]. Over recent decades, the incidence and prevalence of IBDs have increased not only in the West but also in Asia as industrialization and westernization have progressed [4]. Although the etiology of IBDs is not understood yet, the main etiology of IBDs has been considered as a complex interaction of diverse causes such as environmental, genetic, or microbial factors and immune responses [5]. In terms of pathogenesis, tumor necrosis factor-α (TNF-α) has been shown to play a crucial role in chronic intestinal inflammation. Therefore, anti-TNF-α agents are widely used in immunosuppressive drug therapy along with conventional anti-inflammatory drugs such as sulfasalazine and 5-aminosalicylic acid (5-ASA) [6]. However, not only the side effects but also the high cost of recent treatments for IBDs are a burden to patients [7]. Concurrently, existing therapies have not shown full remedial value in one-third of IBDs patients [8]. Thus, the development of alternative treatments and/or effective remission of IBDs are required.

Soybean-derived isoflavones act as phytoestrogens in mammals and have been evaluated for health benefits such as antioxidant, antimicrobial, and anti-inflammatory activity [9,10,11,12]. Over several decades, various studies have reported that soybean isoflavones, such as genistein, glycitein, and daidzein, are beneficial and potential therapeutic compounds for inflammation-related diseases [13,14]. Each isoflavone consists of up to four different forms: aglycone, non-conjugated glycoside, acetyl-glycoside, and malonyl-glycoside [15]. Several animal and human studies have suggested that glycoside isoflavones could be more efficient in terms of bioavailability than aglycones [16,17]. Setchell et al. reported that the bioavailability of isoflavones was higher from glycosides than from aglycones when measuring the area under curve in plasma [16]. Moreover, the plasma concentration of equol, a metabolite of daidzein, was significantly higher in an isoflavone glycosides intake group compared with an aglycones intake group in American women [17].

Soybean-derived isoflavone glycosides (SIFs) were isolated from a soybean hypocotyl fraction containing 51.94 w/w% (22.00 w/w% as aglycone equivalents) of isoflavone glycosides, where the three major malonyl-glycosides of glycitin, daidzin, and genistin accounted for approximately 66.5% of total isoflavones (described in the Materials and Methods). It has been reported that SIFs have beneficial effects for menopausal hot flushes [18] and an inhibitory effect on contact hypersensitivity in mice [19]. Moreover, a recent study demonstrated that SIF inhibited mRNA processing and may be a potential anticancer candidate [20]. However, whether SIFs exert anti-inflammatory effects and/or mechanism on the intestines and SIF immune cells including macrophages remains unclear.

For identifying the pathogenesis and/or underlying mechanism of IBDs, dextran sodium sulfate (DSS)-induced IBDs model, which is widely used due to its simplicity and reproducibility. DSS-induced murine colitis model is associated with severe epithelial damage and a robust inflammatory response in the colon [21]. In a mouse model, DSS-induced acute colitis symptoms saw body weight loss, diarrhea, and rectal bleeding [21,22]. Histologically, it was revealed that symptoms included colon length shortening, mucosal and submucosal ulceration/erosion, loss of crypt structure, and rapid increasing of the immune cell infiltration [23]. In IBDs and experimental animal models, intestinal immune cells such as macrophages and intestinal epithelium cells, produced large amounts of chemokines and cytokines, including TNF-α, interleukin (IL)-1β, IL-6, and monocyte chemoattractant protein-1 (MCP-1), both proceeding to inflammatory processes leading to epithelial barrier defects and tissue damage [3,24,25,26].

Although the inflammation response is essential to protect from infection or tissue damage, over-activation or unnecessary maintenance of this process may cause severe damage [26]. Macrophages play key roles in the inflammatory response and host defense. Activation of macrophages by stimuli, such as lipopolysaccharide (LPS), enhances the production of inflammatory cytokines and mediators such as TNF-α, ILs, nitric oxide (NO), and prostaglandins (PGs) [27]. Several previous studies reported that excessive generation of NO plays a critical role in the inflammatory process and pathogenesis of several physiological conditions, including septic shock, atherosclerosis, and carcinogenesis [28,29]. The expression of inducible nitric oxide synthase (iNOS) promotes excessive NO production [30]. Moreover, PGs also act as inflammatory mediators to induce fever, pain, and other symptoms [31]. Prostaglandin E2 (PGE2) is an important inflammatory mediator produced by cyclooxygenase-2 (COX-2). Therefore, during inflammation process, inhibition of the enzyme activities of iNOS and/or COX-2 are an important approach to preventing inflammation-related diseases.

iNOS and COX-2 expression in macrophages is mainly regulated at the transcriptional level. Nuclear factor-kappa B (NF-κB) is an important immunoregulatory gene involved in inflammatory responses, including iNOS and COX-2 regulation [32]. NF-κB exists in the cytoplasm in an inactive state in complex with inhibitory kappa B (IκB) proteins. Upon NF-κB activation with stimuli, such as LPS, it is released from IκB and translocated into the nucleus, where it binds the promoter of target genes and activates transcription [33,34].

The present study was designed to evaluate the anti-inflammatory effects of SIF, water-soluble dietary soybean isoflavones, on DSS-induced colitis in a C57BL/6 murine model and LPS-induced inflammatory mediators and pro-inflammatory cytokines, including their possible regulatory mechanisms in RAW264.7 murine macrophage cell line.

## 2. Materials and Methods

### 2.1. Materials

The following monoclonal or polyclonal antibodies (mAbs or pAbs) were purchased from Cell Signaling Technologies (CST, Danvers, MA, USA) and used for immunoblotting: rabbit anti-iNOS mAb (#13,120) and anti-COX-2 mAb (#12,282), NF-κB pathway sampler kit (#9936) containing two anti-mouse mAbs against I kappa B kinase (IKK) α, IκBα and five anti-rabbit mAbs against IKKβ, phospho-IKKα/β, phospho-NF-κB-p65, NF-κB-p65. Rabbit anti-GAPDH mAb (#5174) was used as loading control. Horseradish-peroxidase-conjugated anti-rabbit or anti-mouse IgG mAbs (CST) were used to visualize the proteins on the immunoblots. The water-soluble soybean-derived isoflavone glycosides (SIFs; Soyaflavone HG; Lot. 181124-02) were donated by Fuji Oil Co, Ltd. (Tokyo, Japan). The total SIF content was 51.94% (w/w) isoflavones (22.00%, as aglycone equivalents), the isoflavone composition as aglycone equivalents was described as Table 1. The proximate composition of SIF was (w/w%): moisture, 2.4%; protein, 12.2%; fat, <0.1%; minerals: 3.4%; carbohydrate, 82.0%. The energy value of the compound was 377 kcal per 100 g. dextran sulfate sodium salt (DSS; 0216011050, colitis grade) was purchased from MP Biomedicals (Santa Ana, CA, USA). Lipopolysaccharide (LPS; from E. coli serotype O55:B5, phenol extraction) was obtained from Sigma Aldrich (Darmstadt, Germany).

### 2.2. Animals and DSS-Induced Colitis Model

Six-week-old male C57BL/6N mice were purchased from Nihon SLC (C57BL/6NCrslc, Shizuoka, Japan). Two weeks after arrival, we divided the mice randomly into five groups (n = 4). The control group received drinking water during the experimental period. The second group was administered drinking water with 2.5% DSS only during DSS induction periods. The other three groups were pre-treated with SIF (0.25%, 0.5%, and 1% [w/v]) in drinking water for 2 weeks before DSS-induction periods. Mice were monitored daily using measures of body weight, rectal bleeding, and water consumption through the DSS treatment period. At the end of the protocol, animals were sacrificed by cervical dislocation. Mice were kept in specific-pathogen free (SPF) conditions in a temperature-controlled room with a 12 h light and 12 h dark cycle and had free access to diet and drinking reverse osmosis (RO) water. All mice experiments were approved by the National Center for Geriatrics and Gerontology (NCGG) Animal Care and Use Committee (Animal-30-28-R3).

### 2.3. TUNEL Assay

A study for terminal deoxynucleotidyl transferase dUTP nick end labeling (TUNEL) assay was determined with using an ApopTag^®^ Red in situ Apoptosis Detection Kit (S7165, Millipore, MA, USA) in accordance with the manufacturer’s protocol. In brief, paraffin-embedded colonic tissue samples were sectioned (4 µm). Tissue sections were deparaffinized and pre-treated with freshly diluted protein digesting enzyme for 15 min at room temperature. Next, tissue sections were incubated in equilibration buffer for 10 sec then incubated in a labeling solution of working strength TdT enzyme at 37 °C for 1 h. After incubation, sections were incubated in stop/wash buffer for 10 min and washed with PBS three times. For the detection of DNA fragmentation, sections were incubated in working strength anti-digoxigenin conjugate (rhodamine) for 30 min in the dark at room temperature. After four washes with PBS, the cell nuclei were counterstained with DAPI (0.5 µg/mL). Images of section were obtained using a BZ-X710 All-in-one inverted fluorescence microscope (Keyence, Osaka, Japan) with 4× and 20× objective lenses and BZ-X filter TRITC (ex/em 545/605), BZ-X filter DAPI (ex/em 360/460). The BZ-X analyzer software (Keyence, Osaka, Japan) was used for image analyzing.

### 2.4. Histology

The resected mice colon tissues were washed with cold PBS followed by immediate fixation in 4% PFA then stored at 4 °C overnight. For histopathological analysis, paraffin-embedded tissue sample were sectioned (4 µm) and stained with hematoxylin and eosin (H and E). All images of sections were obtained using a BZ-X710 All-in-one inverted fluorescence microscope (Keyence, Osaka, Japan) with 10× and 20× objective lenses. The images were analyzed using BZ-X analyzer software (Keyence) and ImageJ software (NIH, Bethesda, MD, USA). For the immunohistology of F4/80 positive cells in colon tissues, frozen OCT-embedded tissue samples were sectioned (8 µm) at −20 °C then post-fixation with 4% PFA for 10 min. Permeabilization was performed with 0.5% triton x-100 in PBS with 15 min. The sections were blocked by 10% goat serum (Nichirei Bioscience, Tokyo, Japan) for 30 min. Immunostaining of F4/80 was performed with APC conjugated F4/80 antibody (123115, BioLegend, San Diego, CA, USA) at 4 °C, overnight. Sections were mounted with Prolong gold antifade mountant with DAPI (Thermo Scientific, Waltham, MA, USA). The images were captured with LSM-780 confocal laser microscopy (Carl Zeiss, Jena, Germany). The excitation wavelength was set with 633 nm laser for APC detection and 405 nm laser for DAPI. The images were analyzed using Zen software (Carl Zeiss).

### 2.5. Histological Score

H and E-stained colon sections were assessed by a pathologist in a blinded study using a microscopic scoring system based on the following parameters. Severity of inflammation was scored as follows: 0, rare inflammatory cells in the lamina propria (<10%); 1, mild (10–25%); 2, moderate (26–50%); and 3, severe (>51%). The extent of inflammatory cell infiltration was scored as follows: 0, normal; 1, mucosal infiltration; 2, submucosal infiltration; or 3, transmural infiltration. Crypt damage or erosion was scored as follows: 0, normal; 1, mild; 2, moderate; or 3, severe.

### 2.6. Quantitative Real-Time PCR

Total RNA was extracted from mouse colon tissues or cultured cells using TRI reagent (Molecular Research Center, Cincinnati, OH, USA). Any genomic DNA contaminating the sample was digested with DNase I (Qiagen, Hilden, Germany). Further purification was performed using a RNeasy Cleanup Kit (Qiagen). One microgram of total RNA was reverse-transcribed using a ReverTra Ace qPCR RT Kit for reverse transcription polymerase chain reaction (RT-PCR; Toyobo, Tokyo, Japan). Quantitative PCR was performed using Thunderbird SYBR qPCR Mix (Toyobo) or Thunderbird Probe qPCR Mix (Toyobo). Gene-specific primer sets were obtained from SYBR green gene expression assays for mouse iNOS (NOS2; forward, TTCTCAGCCCAACAATACAAGA; reverse, GTGGACGGGTCGATGTCAC), MCP-1 (MCP1/CCL2; forward, AGGTCCCTGTCATGCTTCTG; reverse, TGAGTAGCAGCAGGTGAGTG), GAPDH (forward, CTACTGGCGCTGCCAAGGC; reverse, GTGGGTGTCGCTGTTGAAGTC) (FASMAC, Kanagawa, Japan). Gene-specific probes were obtained from TaqMan gene expression assays for TNF-α (Mm00443258_m1), IL-1β (Mm00434228_m1), IL-6 (Mm00446190_m1) and GAPDH (Mm99999915_g1) (Applied Biosystems, Foster City, CA, USA). Each sample was analyzed in duplicate. Amplification and real-time detection were performed using Pico-real (Thermo Scientific). The expression levels of the target genes were normalized to those of mouse glyceraldehyde-3-phosphate dehydrogenase (GAPDH) as an endogenous control.

### 2.7. Cell Culture

RAW264.7 murine macrophage cells (originated from ATCC TIB-71) were a generous gift from Dr. Nakanishi (National Center for Geriatrics and Gerontology; NCGG, Aichi, Japan) and cultured at 37 °C in 5% CO_2_ in Dulbecco’s modified Eagle’s medium containing 10% fetal bovine serum (FBS), 100 µg/mL of penicillin, and 100 µg/mL of streptomycin. For all experiments, cells were grown to 80–90% confluency.

### 2.8. WST-1 Cell Viability Assay

Cells were seeded in 96-well plates. Confluent cells were deprived of serum for 12 h and were then treated with SIF (0–5 mg/mL) only or treated with SIF (0–3 mg/mL) after treatment with 0.3 µg/mL LPS for 24 h. Viable adherent cells were stained with Proliferation Reagent WST-1 (Roche Diagnostics GmbH, Penzberg, Germany) for 30 min at 37 °C in 5% CO_2_. The WST-1 assay is based on the reduction of WST-1 by viable cells and is suitable for measuring cell proliferation, cell viability, and cytotoxicity. The reaction produces a soluble formazan salt. Absorbance was assayed at 440 nm using a microplate reader (Spectramax m5; Molecular Devices, San Jose, CA, USA).

### 2.9. Measurement of NO

Confluent RAW264.7 cells were pre-incubated in serum-free medium at 37 °C for 12 h, then pre-treated with SIF (0–1 mg/mL) 12 h prior to LPS (0.3 µg/mL) exposure for up to 48 h. The level of NO production was monitored by measuring the nitrite level in the culture medium using a Griess reagent kit (Thermo Scientific, Waltham, MA, USA). The absorbance was measured at 540 nm using a Spectramax m5 (Molecular Devices).

### 2.10. Measurement of PGE2

Cell culture supernatants were measured with a Parameter Prostaglandin E2 Assay kit (R&D Systems, MN, USA) in accordance with the manufacturer’s instructions. RAW264.7 cells were incubated in serum-deprived medium for 12 h then pre-treated with SIF for 12 h. Supernatants were collected 48 h after LPS treatment. Collected supernatants were diluted 40-fold and absorbance was measured at 450 nm with a Spectramax m5 (Molecular Devices).

### 2.11. Immunoblotting Assay

Harvested cells were lysed in M-PER reagent (Life Technologies, Carlsbad, CA, USA) containing protease and de-phosphorylation inhibitors (Wako Pure Chemical, Osaka, Japan). Debris in the supernatant was removed by centrifugation at 12,000× *g* for 15 min. Protein samples (5 µg) were separated on 5–20% ePAGEL Bis-Tris gels (ATTO, Tokyo, Japan), transferred to PVDF membranes, and blotted using standard methods. Protein bands were visualized using an enhanced chemiluminescence system (Thermo Fisher Scientific). To detect phosphorylated and total form of Abs, membranes were stripped with WB stripping solution strong (Nacalai Tesque, Kyoto, Japan) in accordance with the manufacturer’s instructions. The immunoblotting images were taken with a LAS-4000mini (Fuji Film, Tokyo, Japan). Data were analyzed using Multi Gauge software (Fujifilm, Kyoto, Japan).

### 2.12. Luciferase activity ASSAY

The cells were seeded at 2 × 104 cells in 24-well plates and transfected on the following day. A dual luciferase reporter assay system (Promega, Madison, WI, USA) was used to measure the promoter activity. The pGL3-basic plasmid (Promega) containing the mouse IL-6 promoter region, including the wild-type (mIL6-NF-κB-WT-Luc) or mutated NF-κB binding site (mIL6-NF-κB-Mut-Luc), were a generous gift from Dr. M Sugimoto. (NCGG) [35]. Briefly, the cells were transiently co-transfected with 200 ng of mIL6-NF-κB-WT-Luc or mIL6-NF-κB-Mut-Luc and 20 ng of the pRL-TK plasmid (Renilla luciferase expression for normalization) (Promega) using X-treme Gene 9 (Roche). Cells were then pre-incubated with SIF (0–1 mg/mL) for 12 h and exposed to LPS for 4 h. Firefly and Renilla luciferase activities in the cell lysates were measured using a luminometer (Promega). The relative luciferase activity was calculated by normalizing the firefly luciferase activity to that of Renilla luciferase.

### 2.13. Confocal Microscopy

For confocal microscopy, RAW264.7 cells were pre-incubated with or without SIF prior to treatment with 0.3 µg/mL LPS for 30 min. The cells were then fixed in 4% paraformaldehyde at room temperature for 10 min. For immunostaining, the cells were blocked with 10% goat serum (Nichirei Bioscience, Tokyo, Japan) for 1 h. The cells were immunostained with mouse NF-κB-p65 mAb at room temperature for 1 h. After three washes in PBS, the cells were stained with goat anti-mouse Alexa Fluor 488-labeled secondary IgG Ab (Life Technologies, Carlsbad, CA, USA) at room temperature for 1 h. The nuclei were stained with DAPI (Life Technologies). Immunofluorescence images were obtained using a confocal laser scanning microscope (LSM-780; Carl Zeiss, Jena, Germany). The images were processed using Zen software (Carl Zeiss) or ImageJ software (NIH).

### 2.14. Statistics

All data are expressed as the means ± standard deviation (SD). Significance was assessed using one-way ANOVA, followed by Tukey’s post hoc test with a single pooled variance for multiple comparison. Two-way ANOVA followed by Sidak’s multiple comparison test for body weight loss comparison. Statistical analysis was performed using GraphPad Prism 7 software (GraphPad Software, San Diego, CA, USA). Significance was accepted at values of *p* < 0.05.

## 3. Results

### 3.1. SIF Alleviates Symptoms of DSS-Induced Colitis in C57BL/6N Mice

**Figure 1:**
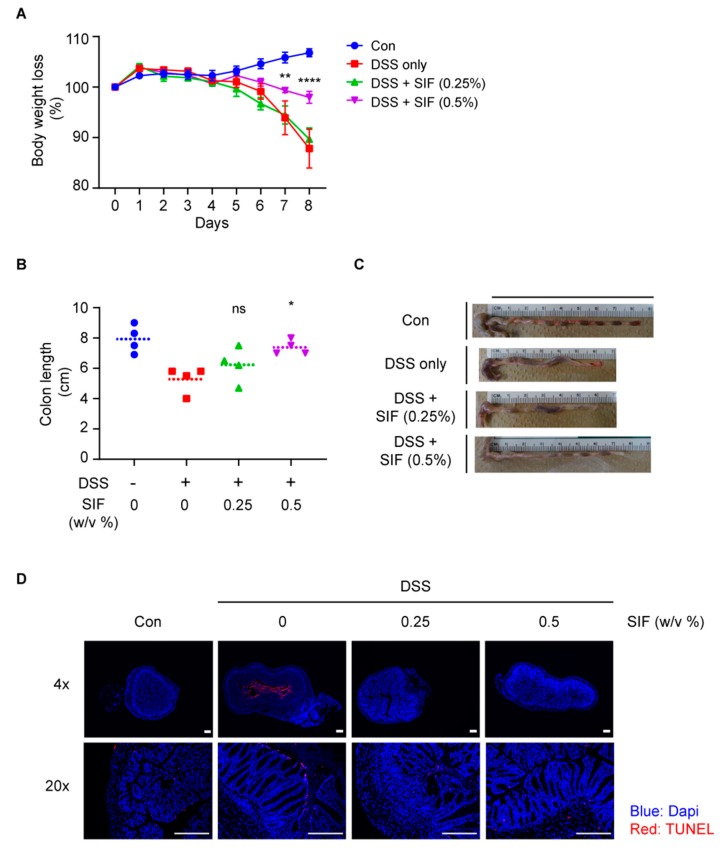
Effect of SIF treatment on dextran sodium sulfate (DSS)-induced colitis in C57BL/6N mice. Oral administration of SIF was started 2 weeks prior to DSS treatment. (**A**) The rate of body weight alteration in control and DSS-induced colitis mice with (w/w) or without (w/o) SIF (0.25 and 0.5 w/v% in drinking water) treatment. The difference between the 0.5% (w/v) SIF-treated group and non-treated group was significant from 7 days. Data represent the means ± SE (n = 4, significance was compared with the DSS-only treatment group. ** *p* < 0.01, **** *p* < 0.0001). (**B**,**C**) Measurement and representative images of colon length from experimental mouse groups. Individual colon lengths were plotted. Dashed lines represent the means of each group (n = 4, significance was compared with the DSS-only treatment group, * *p* < 0.05, ns indicates not significant). (**D**) TUNEL-positive and DAPI-stained DSS-induced colon tissues. Representative images of TUNEL-labeled (rhodamine, red) apoptotic cells counter-stained with DAPI (blue) as described in the Materials and Methods. Arrows denote apoptotic cells in colon. Scale bar for the image indicates 200 µm.

To clarify the physiological effect of SIF-mediated anti-inflammatory effect in vivo, a DSS-induced colitis model was used in C57BL/6N mice. The treatment effect of SIF on this DSS-induced colitis murine model was determined using body weight alteration, colon length shortening, and the pathohistological diagnosis. In this DSS-induced colitis model, SIF mitigated body weight loss and colon length shortening by DSS administration (Figure 1A). Compared with the DSS-only treatment group which showed body weight loss from day 2, the 0.5% SIF pre-treatment group demonstrated protected effect on body weight loss from day 7 to day 8. Moreover, we measured colon length immediately after dissection of mouse colon and found that SIF significantly reduced colon length shortening by DSS administration (Figure 1B,C). In addition, the cellular apoptosis in the colon tissues was detected using TUNEL assays (Figure 1D). DSS-treatment led to increased numbers of apoptotic cells in the crypts, especially the apical side of the epithelium that faces the lumen. The number of DSS-induced apoptotic cells were suppressed by the SIF treatment.

### 3.2. SIF Mitigates the Histopathology of DSS-Induced Colitis

**Figure 2:**
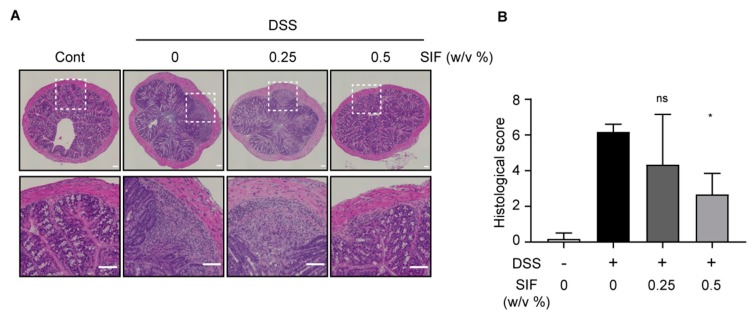
Effect of SIF on histology in DSS-induced murine colitis. (**A**) Hematoxylin and eosin (H and E) stained colon tissue section from control and DSS-colitis mice with or without SIF treatment. Representative Images were obtained using a BZ-X710 microscope (10× magnification) with automated image stitching. Scale bar for the image indicates 100 µm. (**B**) Histological scores of colon paraffin-embedded sections of DSS-induced colitis mice with or without administration of SIF. Data represent means ± SD. Significance was compared with the DSS-only treatment group, * *p* < 0.05, ns indicates not significant. Histological scoring system was as described in the Materials and Methods.

As shown in Figure 2A, representative images of H and E-stained colon sections showed that DSS administration resulted in disruption of colon crypt structure and severe histological inflammation including immune cell infiltration whereas the control group appeared morphologically normal in colon tissues. However, H and E staining histology performed in the SIF treatment group (0.5%, w/v) showed recovery of colon tissue deterioration and colitis severity compared with the DSS-only treatment group. Histological score measurements were performed using light microscopy by a pathologist. The grading of histological score was as described in the Materials and Methods. The pathohistological scores were significantly lower in the SIF pre-treated group than in the DSS-only treatment group (Figure 2B).

### 3.3. SIF Suppresses mRNA Expression of Pro-Inflammatory Cytokines and Inflammatory Enzymes in DSS-Induced Colitis Tissue

**Figure 3:**
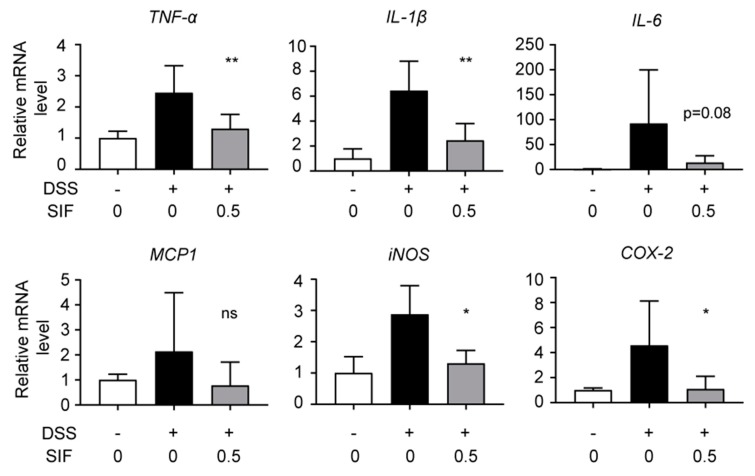
Effect of SIF on the mRNA expression levels of inflammatory cytokines and chemokines in DSS-induced colitis tissue. Total RNA was extracted from colon tissues of untreated and DSS-treated mice as described in the Materials and Methods. The relative mRNA levels of *TNF-α*, *IL-β*, *IL-6*, *MCP1*, inducible nitric oxide synthase (*iNOS*), and cyclooxygenase-2 (*COX-2*) were measured using quantitative RT-PCR assays. Data represent the means ± SD. (n = 4 or 7, ratio to control group. Significance was compared with the DSS-only treatment group, * *p* < 0.05, ** *p* < 0.01, ns indicates not significant). The mRNA expression level of mouse glyceraldehyde-3-phosphate dehydrogenase (*GAPDH*) was used as a housekeeping gene.

Next, to confirm the anti-inflammatory effect of SIF on the increased DSS-induced colitis model, we investigated the mRNA expression levels of inflammatory cytokines such as *TNF-α*, *IL-1β*, *IL-6*, *MCP1* and inflammatory enzymes *iNOS* and *COX-2* in colon tissues assessed by quantitative RT-PCR. As shown in Figure 3, SIF significantly suppressed mRNA expression levels of *TNF-α*, *IL-1β*, *iNOS*, and *COX-2* induced by DSS administration.

### 3.4. SIF Decreases Infiltration of F4/80-Positive Immune Cells in DSS-Induced Colitis Mice

**Figure 4:**
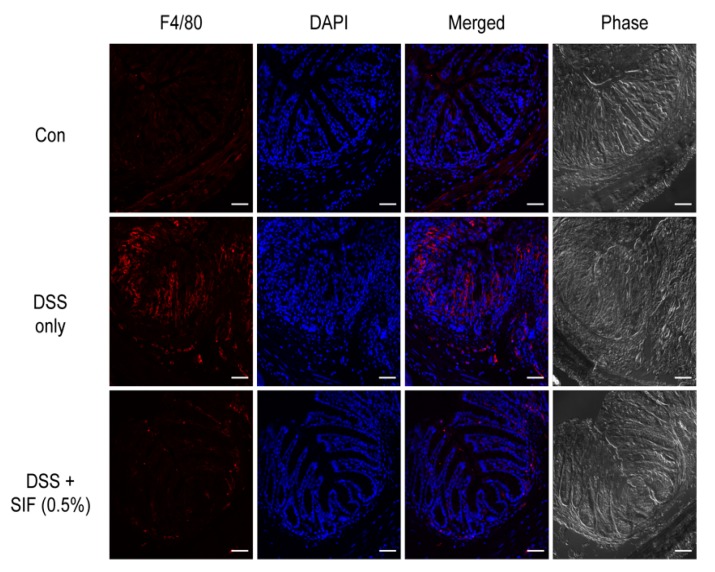
Effect of SIF on the number of F4/80-positive macrophages in DSS-induced colitis mice. Confocal microscopy images of OCT embedded frozen colon tissue stained with APC conjugated anti-mouse F4/80 antibody (APC, red) and nuclei (DAPI, blue). Microscopic images of F4/80, nuclei, merged signals (APC and DAPI), and phase contrast are shown from the left to right panels, respectively. All images were taken using an LSM-780 microscope (Carl Zeiss). Original magnification: ×20. Scale bars of the images represent 50 µm.

To investigate immune cell infiltration in DSS-induced colitis, we conducted immunofluorescence staining of colonic mucosa (Figure 4). F4/80-positive cells were drastically increased in the lamina propria and submucosal layer in the DSS-only treatment group compared with the control group. In the 0.5% SIF treatment group, however, significant differences were observed in the number of F4/80-positive cells. This result was in agreement with the histological colitis change.

### 3.5. SIF Inhibits NO and PGE2 Production Via iNOS and COX-2 Expression in LPS-Activated RAW264.7 Macrophages

**Figure 5:**
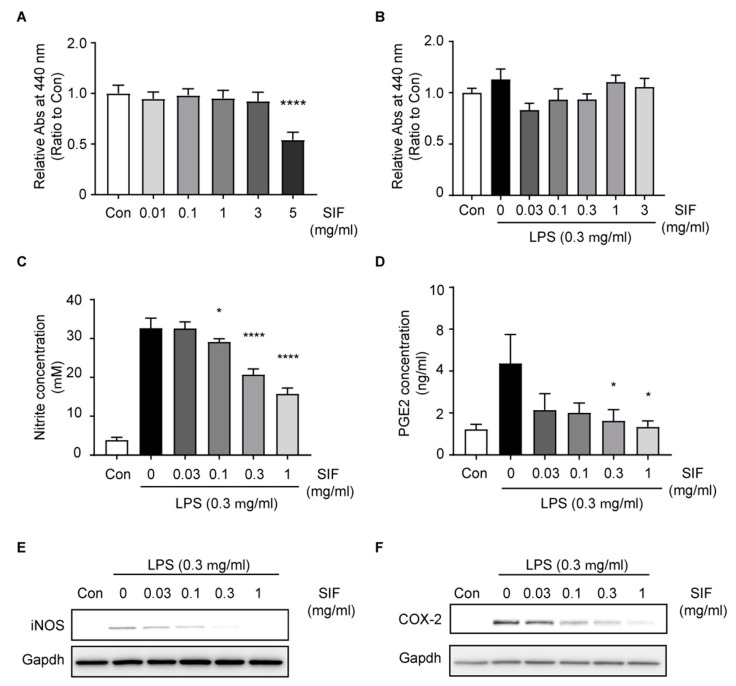
Effect of SIF on nitric oxide (NO) and prostaglandin E2 (PGE2) production via iNOS and COX-2 expression in LPS-induced RAW264.7 macrophages. (**A**) Effect of SIF on cell viability of RAW264.7 macrophage cells. RAW264.7 cells were pre-activated with serum deprivation for 12 h, after which the cells were treated with different concentrations of SIF (0–5 mg/mL). Each value indicates the mean ± S.D. (n = 8, significant compared with the control (Con) group). (**B**) The cell viability of RAW 264.7 macrophages treated with different concentrations of SIF (0–3 mg/mL) and lipopolysaccharide (LPS) (0.3 µg/mL) for 24 h. Data indicates the mean ± S.D. (n = 8, significant compared with Con group). (**C**) RAW264.7 cells were pre-incubated with SIF at different concentrations (0–1 mg/mL), LPS (0.3 µg/mL) was added during the 48 h incubation period. NO production was determined by measuring the nitrite concentration in the culture media by the Griess reaction. Data indicates the mean ± S.D. (n = 4, significant compared with the LPS-only treatment group, * *p* < 0.05, **** *p* < 0.0001). (**D**) Inhibitory effects of SIF on PGE2 production was measured using the parameter PGE2 assay. Data represent the mean ± S.D. (n = 8, significant compared with the LPS-only treatment group. * *p* < 0.05). (**E**,**F**) Effects of SIF pre-treatment on LPS-induced iNOS and COX-2 protein expression during a 24 h incubation period. Protein expression levels of iNOS and COX-2 were detected using immunoblotting assays. GAPDH expression was used as a loading control.

To understand the anti-inflammatory effect and mechanism underlying SIF on immune cells, we determine the anti-inflammatory effect of SIF in an in vitro experimental model with using an LPS-activated RAW264.7 murine macrophage cell line. To confirm the anti-inflammatory effects of SIF, we evaluated its cellular toxicity, and then assessed its effects on cell viability with a WST-1 assay. RAW264.7 cells were incubated for 24 h with SIF at a wide range of concentrations (0–5 mg/mL). We found that treatment with SIF of up to 3 mg/mL did not alter the viability of RAW264.7 macrophages (Figure 5A). We performed the cell viability assay with RAW264.7 macrophages incubated with SIF at varying concentrations (0–3 mg/mL) that were then exposed to LPS (0.3 µg/mL) for 18 h. There was no significant difference in cell viability at concentrations of SIF of up to 3 mg/mL (Figure 5B). Thus, we treated cells with SIF in the concentration range of 0.03–1 mg/mL during subsequent experiments.

To clarify the inhibitory effects of SIF on NO production, we measured nitrite levels and PGE2 concentration in culture media after 48 h exposure to LPS (0.3 µg/mL) in RAW264.7 macrophages using Griess reagent and a PGE2 parameter assay. LPS-stimulated RAW264.7 macrophages had an increased nitrite concentration and in the cultural media compared with the control group. However, pre-treatment with SIF reduced the LPS-induced nitrite concentration in a concentration-dependent manner (Figure 5C). Likewise, as shown in Figure 5D, the LPS-induced PGE2 concentration was decreased by pre-treatment with SIF at concentrations of 0.3 and 1 mg/mL. Next, we performed immunoblotting assays to evaluate the effects of SIF on the expression of iNOS and COX-2, leading to the modulation of LPS-stimulated NO and PGE2 production (Figure 5E,F). The protein expression levels of LPS-induced iNOS and COX-2 were decreased by pre-treatment with SIF in a concentration-dependent manner. This indicated that SIF inhibits excessive NO and PGE2 production through iNOS and COX-2 expression in LPS-activated RAW 264.7 macrophages.

### 3.6. Effects of SIF on Pro-Inflammatory Cytokines and Chemokines in LPS-Activated RAW264.7 Macrophages

**Figure 6:**
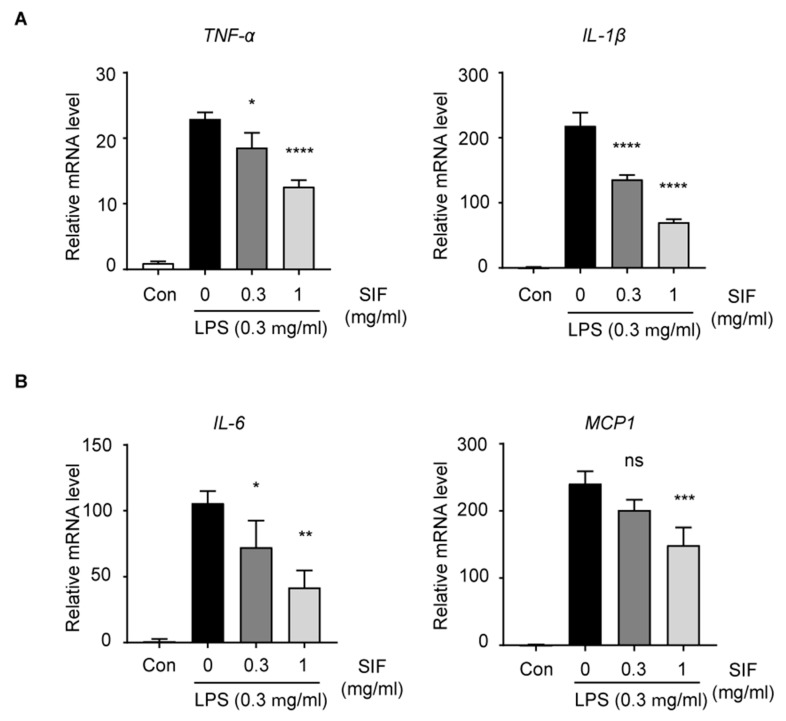
Effects of SIF on pro-inflammatory cytokines and chemokines in vitro. (**A**) Relative mRNA expression levels of *TNF-α* and *IL-1β* after 1 h exposure to LPS in RAW264.7 macrophages were measured using quantitative PCR assays. (**B**) Relative mRNA expression levels of *IL-6* and *MCP-1* after 12 h exposure to LPS. Each value indicates mean ± S.D. (n = 3, ratio to Con group, significant compared with the LPS-only treatment group, * *p* < 0.05, ** *p* < 0.01, *** *p* < 0.001, **** *p* < 0.0001, ns indicates not significant). The mRNA expression level of *GAPDH* was used as a housekeeping gene.

Next, we investigated the effects of SIF treatment on LPS-induced mRNA expression of pro-inflammatory cytokines and chemokines using quantitative RT-PCR. We found that LPS stimulated mRNA gene expression in RAW264.7 cells up to 24 h (data not shown). Then, we evaluated the effects of SIF on LPS-induced mRNA expression of *TNF-α* and *IL-1β* at 1 h after LPS exposure (Figure 6A), and expression of *IL-6* and *MCP-1* was measured at 12 h after LPS exposure (Figure 6B). As a result, pre-treatment with SIF decreased *TNF-α*, *IL-1β*, *IL-6*, and *MCP-1* expression in LPS-activated RAW264.7 cells.

### 3.7. Effects of SIF on NF-κB Transactivation and LPS-Induced IL-6 Reporter Luciferase Activity

**Figure 7:**
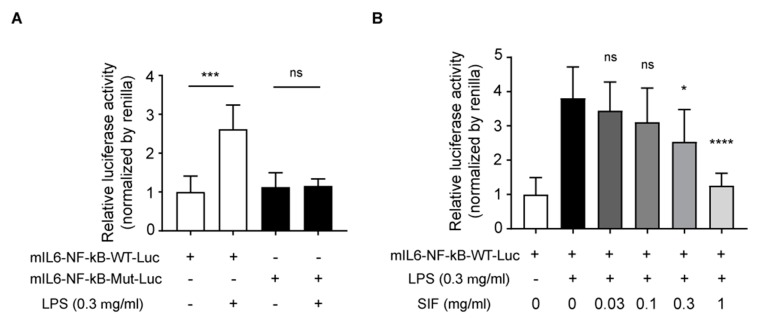
Effects of SIF on NF-κB transactivation and LPS-induced IL-6 reporter luciferase activity. (**A**) RAW264.7 macrophages were transfected with the indicated mIL-6-NF-κB-WT-Luc or mIL6-NF-κB-Mut-Luc plasmid together with pRL-TK Renilla luciferase plasmid (pRL-TK). After 48 h, the cells were deprived of serum for 12 h, then lysates were prepared, and luciferase activity was evaluated 4 h after LPS treatment. Data represent the mean ± S.D. (n = 4, significant compared with each non-treated group, *** *p* < 0.001, ns indicates not significant) (**B**) RAW264.7 macrophages were transfected with mIL6-NF-κB-WT-Luc and pRL-TK Renilla luciferase plasmids (pRL-TK), and 48 h later the cells were pre-treated with SIF for 12 h and LPS (0.3 µg/mL) was added for 4 h. Data represent the mean ± S.D. (n = 8, significant compared with LPS alone, * *p* < 0.05, **** *p* < 0.0001, ns indicates not significant).

NF-κB is a well-known transcriptional factor that regulates inflammatory cytokines and pro-inflammatory proteins [36]. NF-κB activates cells in response to LPS or other inflammatory stimulators, a finding that is related to the transcriptional activation of target genes [36]. Thus, we examined a luciferase activity assay using mIL6-NF-κB-WT-Luc and mIL6-NF-κB-MUT-Luc plasmids containing the luciferase structural gene and mouse IL-6 promoter with wild-type or mutated NF-κB binding site. RAW264.7 macrophages were transiently transfected with each plasmid, then exposed to LPS (0.3 µg/mL) for 4 h. Luciferase activity increased in mIL6-NF-κB-WT-Luc plasmid transfected cells, but there were no significant changes in mIL6-NF-κB-MUT-Luc plasmid transfected cells (Figure 7A). Using the mIL6-NF-κB-WT-Luc plasmid, we next performed a luciferase activity assay to clarify whether SIF affects transcriptional activation via NF-κB binding. As shown in Figure 7B, pretreatment with SIF at a concentration of 0.3 or 1 mg/mL significantly inhibited luciferase activity compared with the LPS-only treatment group.

### 3.8. Effects of SIF Treatment on LPS-Induced NF-κB Nuclear Translocation Via the IKK/IκB Signaling Pathway in RAW264.7 Macrophages

**Figure 8:**
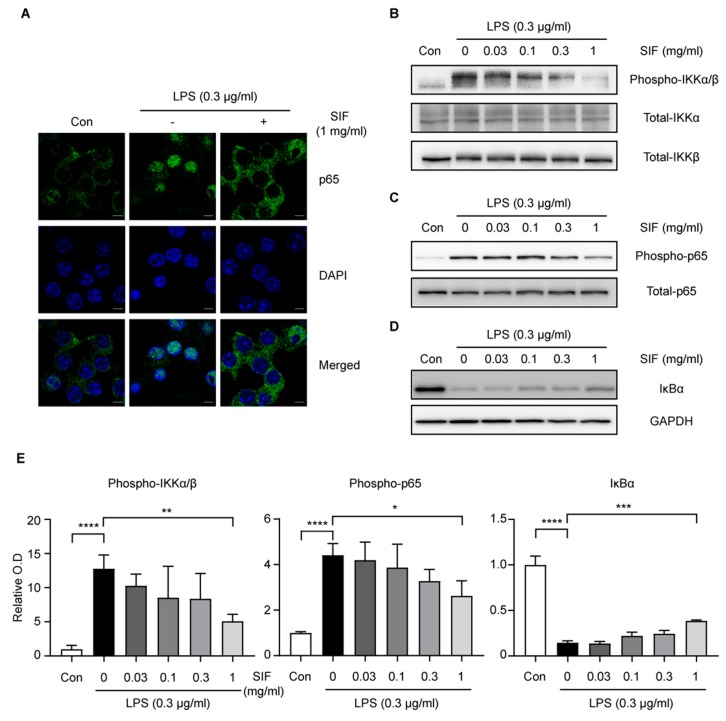
Effects of SIF treatment on LPS-induced NF-κB nuclear translocation via the IKK/IκB signaling pathway. (**A**) Confocal microscope images of RAW264.7 cells stained with anti-NF-κB-p65 antibody (Alexa 488, green) and nuclei (DAPI, blue). RAW264.7 cells were pre-treated with SIF (1 mg/mL) for 12 h prior to LPS treatment for 15 min. Microscopic images of p65, nuclei and merged signals (Alexa 488 and DAPI) are shown in the upper, middle, and lower panels, respectively. All images were taken using an LSM-780 microscope (Carl Zeiss). Original magnification: ×63 with ×3 digital zoom, oil objective. Scale bar for the images indicates 5 µm. (**B**) IKK phosphorylation was detected using an immunoblotting assay with anti-phospho-IKKα/β, total-IKKα and IKKβ monoclonal antibodies, respectively. (**C**) Phosphorylation of p65 was detected using phospho-NF-κB-p65 and NF-κB-p65 antibodies. (**D**) IκB degradation was detected using an anti-IκBα monoclonal antibody. RAW264.7 cells were pre-treated with SIF at different concentrations (0–1 mg/mL) for 12 h. Total cell lysates were collected after 15 min LPS (0.3 µg/mL) treatment. GAPDH was used as a loading control. (**E**) Densitometric quantification of IKK phosphorylation, phosphorylation of p65 and IκB degradation were shown in the left, middle and right panels, respectively. The graph calculated by averaging the results were normalized each internal control from three independent experiments. Data represent the mean ± S.D (ratio to Con, significant compared with LPS alone, * *p* < 0.05, ** *p* < 0.01, *** *p* < 0.001, **** *p* < 0.0001).

NF-κB is generally thought to be constitutively inactive and located in the cytoplasm in most cell types until induced by a stimulus to translocate to the nucleus. The NF-κB heterodimer of p65 and p50 is most commonly found in the canonical NF-κB signaling pathway [37]. Because p65 is a major component of NF-κB activation by LPS in macrophages [38], we examined p65 translocation to the nucleus using an immunofluorescence assay with confocal microscopy. The immunofluorescence assay revealed that nuclear p65 expression increased 30 min after treating RAW264.7 cells with LPS (0.3 µg/mL) (Figure 8A, left and middle lanes). However, pre-treatment with SIF (1 mg/mL) drastically diminished the LPS-induced nuclear translocation of p65 (Figure 8A, right lane). Therefore, SIF inhibited the nuclear translocation of NF-κB p65 that was induced following LPS stimulation.

The translocation of NF-κB to the nucleus is preceded by the phosphorylation, ubiquitination, and degradation of IκB. Furthermore, the IKK-α/β is an upstream kinase of IκB in the NF-κB signal pathway [39]. To determine whether the suppression of NF-κB activation by SIF results from the inhibition of IKK phosphorylation and IκB degradation, we assessed the phosphorylation of IKK and protein levels of IκBα in LPS-stimulated RAW264.7 macrophages. As shown in Figure 8B–E, IKKα/β phosphorylation, IκBα degradation, and phosphorylation of p65 were strongly induced in RAW264.7 macrophages after 15 min of LPS exposure. However, pre-treatment with SIF inhibited the phosphorylation of IKKα/β in RAW264.7 cells in response to LPS treatment (Figure 8B,E left panel). Furthermore, immunoblotting analysis using the IκBα antibody revealed that the expression level of IκBα was suppressed 15 min after LPS treatment (Figure 8D,E right panel). This suppression was also moderated by pre-treating the cells with SIF. Moreover, LPS-induced phosphorylation of p65, activating p65, was attenuated by SIF pre-treatment (Figure 8C,E middle panel). These results suggested that the inhibition of NF-κB activation by SIF is due to the phosphorylation of IKK and prevention of IκBα degradation, and the subsequent activation of p65 phosphorylation. Taken together, these results suggested that SIF inhibits NF-κB activation by regulating a signal transduction mechanism related to the IKK/IκB signaling pathway.

## 4. Discussion

The current therapies for IBDs, including administration of 5-ASA, immunosuppressive drugs, corticosteroids, and TNF-α blockers, are associated with various side effects and high costs for long-term treatment [40]. Thus, it is required to develop effective and safe alternative treatments such as natural substances.

Previous studies reported that soybean-derived isoflavone aglycones such as genistein, daidzein, and glycitein have broad biological activities [9,10,11,12]. However, the chemical and physical characteristics of aglycones include greater hydrophobicity, bitter taste, and less-acceptable odor/flavor than glycosides, which may be the cause of their low bioavailability in vivo and restricted utilization for oral administration [41,42]. Therefore, improvement of the solubility of isoflavone glycosides may be beneficial to enhance their bioavailability and practical use as functional foods.

Glycosides are abundant in dietary soy isoflavones, i.e., as in SIF, and have been reported to exert not only enhanced water solubility but also bioavailability in several disease-related experimental models [18,19,20]. To the best of our knowledge, we have examined for the first time the potential therapeutic effect of SIF against intestinal inflammation status. The present study demonstrated the remission effect caused by SIF after oral administration in DSS-induced murine colitis.

In our experimental model, 2.5% DSS administration induced various symptoms, including body weight loss, diarrhea, colon length shortening, and pathohistological changes in the colon including inflammation and crypt structure distortion. In addition, DSS administration increased the number of apoptotic cells in the colonic mucosa, which might affect colonic epithelial homeostasis. We found that the mRNA levels of pro-inflammatory cytokines and two major target genes, established as anti-inflammatory agents for inflammation related disease, iNOS and COX-2, were highly induced by DSS administrated to colon tissue.

Oral administration of SIF (0.5 w/v%) showed a remission effect on DSS-induced colitis, including preservation of the weight loss, conservation of the colon length, and protection against crypt and mucosal damage. Furthermore, we observed that SIF administration could reduce immune cell infiltration by DSS-treatment in both histology and immunofluorescent staining with the anti-F4/80 antibody. We found SIF administration suppressed the mRNA levels of not only pro-inflammatory cytokines, such as TNF-α, IL-1β, but also iNOS and COX-2 expressions in colon tissue. Based on these results, immune cells may play an important role in DSS-induced colitis via the production of various inflammatory cytokines and mediators.

As in the in vitro study, we used the LPS-activated RAW264.7 murine macrophage cell line, to examine the anti-inflammatory effects of SIF on endotoxin stimulated macrophages. We assessed the effects of SIF on NO and PGE2 production in LPS-activated RAW264.7 macrophages. Moreover, we observed LPS-induced expression of iNOS and COX-2 in RAW264.7 macrophages that were pre-treated with SIF. Our results demonstrated that SIF significantly suppressed the iNOS/NO and COX-2/PGE2 pathways in RAW264.7 macrophages in response to LPS treatment.

Pro-inflammatory cytokines, which include TNF-α and IL-1β, are well known to modulate the expression of iNOS and COX-2 in activated macrophages [32]. We evaluated the effects of SIF on LPS-induced expression of inflammatory cytokines and chemokines, such as TNF-α, IL-1β, IL-6, and MCP-1/CCL2, in RAW264.7 macrophages. We found that SIF inhibited LPS-induced expression of TNF-α, IL-1β, IL-6, and MCP-1 in RAW264.7 macrophages.

NF-κB is a well-known critical transcription factor for inflammatory gene transcription, including for iNOS, COX-2, and IL-6, during the activation of macrophages by LPS [32]. In addition, nuclear translocation of NF-κB is essential for the transcriptional regulation of its target genes [34]. A luciferase activity assay using wild-type and mutant NF-κB binding sequences revealed that SIF inhibits transactivation of IL-6 through a reduction in NF-κB binding in transiently transfected RAW264.7 macrophages in response to LPS treatment. During macrophage inactivation, NF-κB, which forms a complex with IκB, exists in the cytoplasm in an inactive complex form. In the activated state, IκB is phosphorylated and degraded, then the active NF-κB complex, which was released from IκB, translocate into the nucleus [34]. This IκB degradation is modulated by the phosphorylation of IKKα/β in LPS-activated RAW264.7 cells [43]. In the current study, we found that SIF inhibited the degradation of IκB by suppressing the phosphorylation of IKKα/β in LPS-stimulated RAW264.7 macrophages. As a result, SIF decreased LPS-induced NF-κB activation, which was caused by phosphorylation and nuclear translocation of NF-κB.

In conclusion, SIF showed effectiveness in reducing inflammatory responses in vivo and in vitro. Our study demonstrated the inhibitory effects of SIF on the physiological and histological inflammatory response in DSS-induced colitis in vivo and SIF inhibited the activation of macrophages due to its suppression of NF-kB activation in LPS-stimulated RAW264.7 macrophages in vitro. Our findings suggested that SIF is a potential therapeutic compound and/or functional dietary supplement to prevent inflammatory-related diseases including IBDs.

## Figures and Tables

**Table 1:** Isoflavone compositions in soybean-derived isoflavone glycosides (SIFs) ^1^.

Isoflavone Form	Composition ^2^ (w/w%)
Daidzin	22.58
Glycitin	8.08
Genistin	1.19
Malonyl daidzin	37.96
Malonyl glycitin	20.46
Malonyl genistin	8.08
Acetyl daidzin	0.46
Acetyl glycitin	1.12
Acetyl genistin	0.08
Daidzein	ND ^3^
Glycitein	ND
Genistein	ND
Total	100

^1^ SIF contains a total of 51.94% of isoflavones. ^2^ aglycone equivalent. ^3^ ND, not detected.
